# Attachment of Shiga Toxin-Producing *Escherichia coli* (STEC) to Pre-Chill and Post-Chill Beef Brisket Tissue

**DOI:** 10.3390/microorganisms9112320

**Published:** 2021-11-10

**Authors:** Daniel A. Unruh, Bennett C. Uhl, Randall K. Phebus, Sara E. Gragg

**Affiliations:** Department of Animal Sciences and Industry, Food Science Institute, Kansas State University, 1530 Mid-Campus Drive North, Manhattan, KS 66506, USA; unruh.danny@gmail.com (D.A.U.); bennettuhl@gmail.com (B.C.U.); phebus@ksu.edu (R.K.P.)

**Keywords:** Shiga toxin-producing *Escherichia coli*, beef, attachment, stress, temperature

## Abstract

Shiga toxin-producing *Escherichia coli* (STEC) has caused numerous foodborne illness outbreaks where beef was implicated as the contaminated food source. Understanding how STEC attach to beef surfaces may inform effective intervention applications at the abattoir. This simulated meat processing conditions to measure STEC attachment to adipose and lean beef tissue. Beef brisket samples were warmed to a surface temperature of 30 °C (warm carcass), while the remaining samples were maintained at 4 °C (cold carcass), prior to surface inoculation with an STEC cocktail (O26, O45, O103, O111, O121, O145, and O157:H7). Cocktails were grown in either tryptic soy broth (TSB) or M9 minimal nutrient medium. Loosely and firmly attached cells were measured at 0, 3, 5, and 20 min and 1, 3, 8, 12, 24 and 48 h. TSB-grown STEC cells became more firmly attached throughout storage and a difference in loosely versus firmly attached populations on lean and adipose tissues was observed. M9-grown STEC demonstrated a 0.2 log_10_ CFU/cm^2^ difference in attachment to lean versus adipose tissue and variability in populations was recorded throughout sampling. Future research should investigate whether a decrease in intervention efficacy correlates to an increase in firmly attached STEC cells on chilled carcasses and/or subprimals, which has been reported.

## 1. Introduction

The Centers for Disease Control and Prevention (CDC) estimates that *Escherichia coli* O157:H7 causes 96,000 illnesses annually, while an additional 168,000 annual illnesses are caused by the non-O157 *E. coli* serogroups O26, O45, O103, O111, O121, and O145 (known as the “Big Six”) [1]. Beef and beef products are frequently linked to outbreaks of STEC [2], and STEC infections have also been attributed to kidney failure, hemolytic uremic syndrome, as well as death in humans [2,3]. For this reason, the United States Department of Agriculture Food Safety Inspection Service (USDA-FSIS) declared *E. coli* O157:H7 an adulterant in raw ground beef in 1994 and expanded adulterant status to non-intact meat and trimmings in 1999 [4,5]. In 2012, adulterant status was additionally declared for the “Big Six” serogroups in non-intact beef [6]. STEC are associated with beef cattle and are often present at the time of processing [7]. In their 2021 guidelines for minimizing STEC in beef slaughter operations, USDA-FSIS describes how a pre-evisceration wash immediately following hide removal is important for removing bacteria before carcass attachment can occur [8]. In addition to carcass washes and interventions, one way to mitigate the likelihood of STEC presence in beef products is to better understand the attachment of STEC on surfaces during harvest and fabrication (including the beef itself), knowledge of which may translate into new practices for STEC mitigation in beef processing.

Bacterial attachment processes and mechanisms are important concepts for food safety professionals as attachment impacts bacterial presence in the food supply. The process of cellular attachment is complex: interactions at the molecular level are not fully understood and many factors can impact cellular adherence [9]. While studies examining *E. coli* attachment under beef processing conditions are limited [10,11], several studies have investigated a variety of factors and how they impact bacterial attachment. The ability of bacterial cells to attach biotic and abiotic surfaces is influenced by the environment, and can be impacted by nutrient availability, organic material, temperature, as well as physiochemical properties of the food or food-contact surface, among others [2].

Temperature is a major factor, as it has been demonstrated that bacterial growth temperature impacts the growth and survival of STEC in beef [12], while growth media can also impact the ability of *E. coli* to attach to surfaces. As an example, M9 minimal salt medium—a starvation/stress medium—has been used to create an environment that may simulate conditions as they occur in processing and cleaning practices [13]. The meat surface itself—lean or adipose tissue—may impact bacterial attachment. However, the overall research consensus is that bacteria attach equally to lean and adipose tissue, with variation occurring only within 0.5 logs [14,15,16,17,18,19,20].

Cellular appendages and surface proteins can impact bacterial attachment [21,22]. This includes the capsule, fimbriae, outer membrane polymers, S layers [19], adhesins [23], and other attachment organelles. Surface charge and hydrophobicity of the bacterial cell [21,24] also contribute to bacterial attachment. For lean tissue, initial bacterial cell attachment involves interactions with negative charges on the bacterial cell surface [25]. For adipose tissue attachment, bacterial cellular hydrophobicity likely impacts attachment strength and population [25,26].

Much of the research conducted to date has investigated the impact of a variety of factors, such as growth temperature and starvation stress, on the attachment of *E. coli* O157:H7 and other foodborne pathogens to a variety of surfaces, with little research investigating how attachment may differ on a warm versus chilled beef carcass. More recently, Kirsch et al. [22] addressed this knowledge gap, reporting significantly higher attachment of STEC to chilled briskets (4.0 log_10_ CFU/cm^2^) in comparison to non-chilled briskets (3.6 log_10_ CFU/cm^2^); however, the difference in attachment was marginal at 0.4 log_10_ CFU/cm^2^ and did not compare lean and adipose tissues.

Knowledge gaps still exist regarding how STEC attach to lean versus adipose beef tissues and the impact that starvation stress may have on attachment. Therefore, the goal of this study is to investigate how STEC grown in nutrient-dense and nutrient-limited conditions attach to lean and adipose beef tissues that simulate beef harvest (warm carcass) and postharvest (cold carcass) processes.

## 2. Materials and Methods

### 2.1. Experimental Design

This study was designed to simulate warm and chilled beef carcasses at the abattoir using lean and adipose tissue excision samples collected from beef briskets. Prior to inoculation, samples of both tissue types were stored at 30 °C (warm, pre-chill samples) and 4 °C (cold, post-chill samples) to simulate STEC contamination at the abattoir on the pre-chill and post-chill carcass, respectively. Samples were surface-inoculated and populations of loosely attached and firmly attached STEC cells were determined over the course of 48 h. All post-inoculation cold samples were immediately returned to storage at 4 °C while all post-inoculation warm samples were stored at 4 °C after 30 min, which represents the approximate length of time a warm carcass is undergoing harvest prior to entering the cooler. Attachment was determined over the course of 48 h to understand STEC attachment throughout the carcass chilling process.

### 2.2. Beef Sample Preparation

Vacuum-packaged beef briskets were purchased from a local grocer, with one brisket purchased for each replication. A total of six briskets were purchased (three briskets for each media type) from the refrigerated retail-display case from the same grocery store over the course of the study. Upon arrival at the laboratory, refrigerated temperatures were maintained for each brisket until subsequent tissue excision samples were obtained as described below.

Prior to analysis, a thin layer was removed from the brisket surface with a knife in order to remove any residual carcass or fabrication interventions that may interfere with bacterial attachment and/or survival. From this primal cut, 50 cm^2^ samples of adipose (*n* = 20) and lean (*n* = 20) tissue were collected for each replication. Meat samples were separated by lean and adipose tissue and stored in sealed poultry rinse bags at 4 °C for 18–24 h before analysis.

### 2.3. Culture Preparation

Frozen stock cultures stored in tryptic soy broth (TSB; BD Difco, Franklin Lakes, NJ, USA) at −80 °C were used to prepare a cocktail for inoculation. The non-O157 STEC strains used in this study were acquired from Dr. John Luchansky (USDA, ARS, Eastern Regional Research Center, Wyndmoor, PA, USA), but were originally isolated from human samples. The specific STEC strains were as follows: *E. coli* O157:H7 (ATCC 35150) and non-O157 strains O145: NM (83-75), O121:H19 (CDC 97-3068), O111:H- (JBI-95), O103:H2 (CDC 90-3128), O45:H2 (CDC 90-3285), and O26:H11 (H30). These serogroups were chosen specifically because they are associated with 1) serious human clinical disease and 2) causing foodborne infections [27,28,29] across the world, posing a global health threat [27]. TSB-grown STEC were prepared by growing each strain in 9 mL of tryptic soy broth (TSB; Remel, Lenexa, KS, USA) at 37 °C for 18–24 h. M9-grown STEC strains were prepared by transferring a 10 µL loopful from each TSB-grown STEC strain to 9 mL of M9 minimal salts medium (M9; BD Difco, Franklin Lakes, NJ, USA) and grown at 37 °C for 24 h. M9 medium was prepared by supplementing 200 mL M9 minimal salts solution (5×) with 20 mL glucose (20%), 2 mL MgSO_4_ (1.0 M), 0.1 mL CaCl_2_ (1.0 M), and 750 mL deionized water. Following the 24 h incubation, TSB and M9 culture tubes were centrifuged at 5000× *g* for 15 min at 4 °C. For each TSB or M9 tube, the supernatant was discarded and the pellet was re-suspended in 9 mL of 0.1% peptone water (PW; BD Difco, Franklin Lakes, NJ, USA). Resuspended inoculum tubes were combined in equal proportions to prepare an inoculum cocktail, which was diluted in PW to achieve the desired starting titer of ca. 5.0 log_10_ CFU/mL.

### 2.4. Beef Tissue Attachment Assay

On day 0 of the study, all 50 cm^2^ adipose and lean tissue samples were removed from 4 °C storage and randomly assigned to either the warm or refrigerated treatment group. Samples assigned to the warm treatment were heated to a surface temperature of 30 °C (simulating a warm carcass surface temperature during harvest) in an incubator while cold samples remained at 4 °C until inoculation. Immediately prior to inoculation, all samples were placed into a sterile 11.5-inch × 9.5-inch × 2-inch (29.21-cm × 24.13-cm × 5.08-cm) stainless steel pan with a metal grate placed on the bottom, such that samples were not in direct contact with the base of the pan. Samples were surface-inoculated by pipetting 150 μL of the inoculum cocktail onto the meat surface, which was evenly dispersed with an “L-shaped” spreader (Fisher Science, Hampton, NH, USA). Following inoculation, warm samples were stored for 30 min at 30 °C, before being transferred to refrigeration temperature (4 °C), while cold samples were transferred immediately to 4 °C refrigeration.

Sampling of inoculated meat samples was conducted at time points 0 m, 3 m, 5 m, 20 m, 1 h (60 m), 3 h (180 m), 8 h (480 m), 12 h (720 m), 24 h (1440 m), and 48 h (2880 m). Time began when the STEC cocktail had been pipetted and spread onto the tissue surface using the cell spreader. Following inoculation, methods to release loosely and firmly attached cells from the tissue samples were based upon those previously described by Rivas et al. [30], with modifications. Briefly, each 50 cm^2^ sample was transferred into a stomacher bag containing 250 mL of PW and placed into a shaking incubator set at 4 °C and 200 oscillations per minute for 90 s (Excella E24 Incubator Shaker, New Brunswick Scientific, Edison, NJ, USA). This process released loosely attached cells into the PW; therefore, bacterial populations obtained from this PW sample were counted as loosely attached and will be referred to hereafter as “loose.” Following shaking, the 50 cm^2^ sample was aseptically transferred into a new stomacher bag containing 250 mL of fresh PW and homogenized (Stomacher^®^ 400 Circulator, Seward, Bohemia, NY, UK) for 60 s at 230 rpm. This process released firmly attached cells into the PW and bacterial populations obtained from this PW sample were counted as firmly attached and will hereafter be referred to as “firm”. All samples were serially diluted in PW, plated onto MacConkey agar (Remel; Lenexa, KS, USA), and incubated at 37 °C for 18–24 h.

### 2.5. Statistical Analysis

All experimental procedures were replicated three times. Data collected from all three replications were analyzed using the MIXED procedure with LSMEANS statement of Statistical Analysis Software (SAS 9.4; Cary, NC, USA). For each media type, the main effects (sample type, tissue type, temperature, time) and interactions were evaluated for statistical significance at the *p* ≤ 0.05 threshold. Data were analyzed for each media type individually; thus, media type was not included in the statistical model. As all samples were placed into refrigerated storage at 30 min, data for the 0, 3, 5, and 20 min time points (all time points prior to refrigeration) were also analyzed separately to more clearly probe the relationship of initial STEC attachment and product temperature (warm versus cold).

## 3. Results

### 3.1. TSB-Grown STEC

When grown in TSB, *time* was the only significant main effect (*p* < 0.0001). Regarding interactions, sample type (loose versus firm) × time and sample type × tissue type (lean versus adipose) were significant (*p* < 0.0001 for both). Although time by itself was significant, this variable was included in the sample type × time interaction; therefore, data will be shown in regard to sample type and time and will not be shown according to time alone.

Table 1 summarizes how TSB-grown STEC became more firmly attached over time. Prior to 60 min, loosely attached STEC were greater in population than firmly attached cells, with the disparity decreasing over time. At the 60 min time point, the loosely and firmly attached cells are most similar in population: a mere 0.1 log_10_ CFU/cm^2^ difference that is not statistically significant (Table 1; *p* = 0.3778).

Although firmly attached STEC populations were increasing while loosely attached populations were decreasing after 60 min, a statistical difference in population was not detected until 720 min (12 h), when the firmly attached STEC population was 0.2 log_10_ CFU/cm^2^ larger (*p* = 0.0145) than the loosely attached STEC population. At the end of the 2880 min (48 h) storage period, firmly attached STEC were 0.5 log_10_ CFU/cm^2^ greater than loosely attached STEC (*p* < 0.0001). It is important to note that rounding to the nearest 1 decimal place in Table 1 resulted in discrepancies in statistical differences. For example, a 0.2 log CFU/cm^2^ difference was significantly different at 720 min, but was not significant at 180 min.

**Figure 1:**
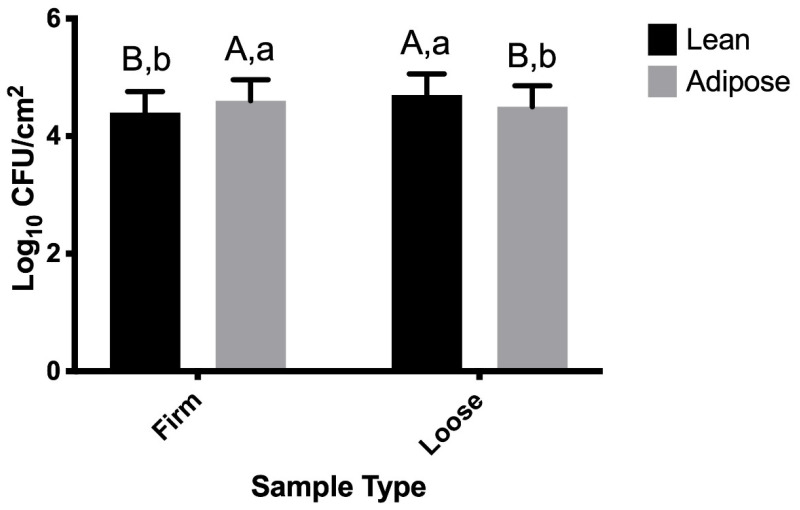
Populations of Shiga toxin-producing *Escherichia coli* (STEC) cells grown in tryptic soy broth (TSB) represent loose and firm attachment to lean and adipose beef brisket tissues. Sample type × tissue type was significant (*p* < 0.0001). As the time × sample type × tissue type interaction was not significant (*p* > 0.05), data for each time point are combined. ^A,B^ Observations with different superscripts within sample type (firm vs. loose) vary statistically (*p* ≤ 0.05). ^a,b^ Observations with different superscripts within tissue type (lean vs. adipose) vary statistically (*p* ≤ 0.05).

The sample type × tissue type interaction that was detected for TSB-grown STEC suggests that STEC attaches differently to lean versus adipose tissue (Figure 1), with a larger population of firmly attached STEC recovered from the adipose tissue.

### 3.2. M9-Grown STEC

Time (*p* < 0.0001) and tissue type (*p* = 0.0134) were the significant main effects. No interactions were significant (*p* > 0.05). As a result, all M9 data will be presented in regard to time and tissue type alone.

**Figure 2:**
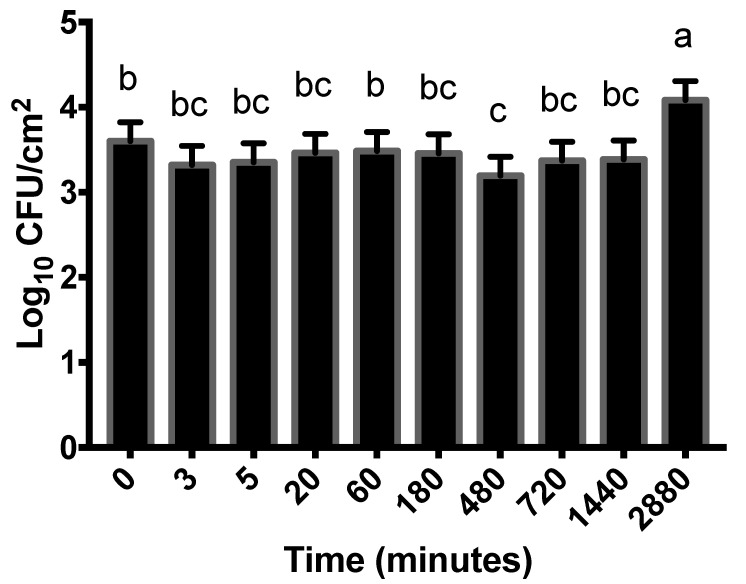
Populations of Shiga toxin-producing *Escherichia coli* (STEC) cells grown in M9 minimal nutrient media attached to lean and adipose beef brisket tissues at each sampling time point. The main effect of time was significant (*p* < 0.0001). As all variable interactions were not significant (*p* > 0.05), all data for each time point are combined into a single observation. ^a,b,c^ Observations with different superscripts vary statistically.

In regard to changes in attachment over time, STEC populations grown in the nutrient-limiting M9 media varied throughout the 2880 min (48 h) storage period (Figure 2). The largest discrepancy (0.9 log CFU/cm^2^) occurred between the 3.2 log CFU/cm^2^ populations at 480 min (8 h) and the 4.1 log CFU/cm^2^ populations at 2880 min (48 h; *p* < 0.0001). At time 0, the STEC population was 3.6 log_10_ CFU/cm^2^, which was similar to populations at all other time points, with the exception of 480 and 2880 min (Figure 2).

**Figure 3:**
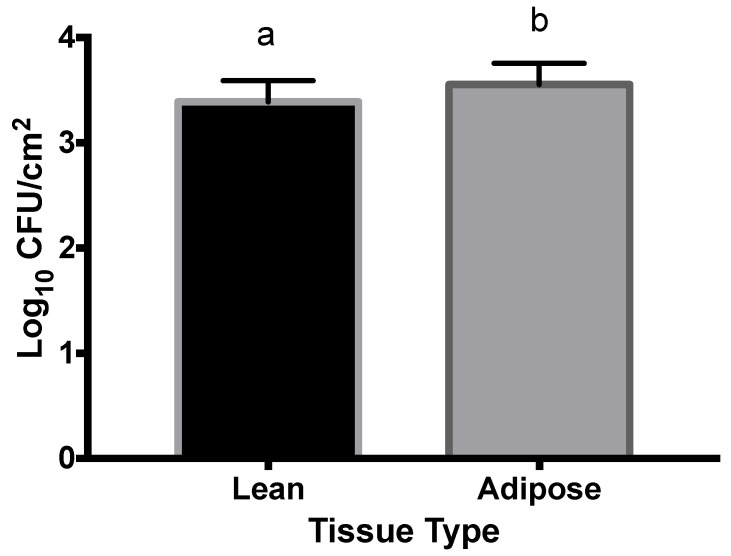
Populations of Shiga toxin-producing *Escherichia coli* (STEC) cells grown in M9 minimal nutrient media attached to lean and adipose beef brisket tissues. The main effect of tissue type (lean vs. adipose) was significant (*p* < 0.0134). As all variable interactions were not significant (*p* > 0.05), all data for each tissue type are combined into a single observation. ^a,b^ Observations with different superscripts vary statistically.

Figure 3 illustrates that a larger population of M9-grown STEC attached to adipose (3.6 log CFU/cm^2^) tissue than lean (3.4 log CFU/cm^2^) tissue (*p* = 0.0134).

### 3.3. Influence of Beef Tissue Temperature on STEC Attachment

Temperature of the beef tissue (warm versus cold) was not a significant variable for either TSB- or M9-grown STEC (*p* > 0.05). The TSB and M9 data collected for all time points prior to refrigeration at 30 min were also statistically analyzed separately from the remaining time points (up to 30 min, or, through the 20 min sampling point as no sampling occurred at 30 min specifically).

**Figure 4:**
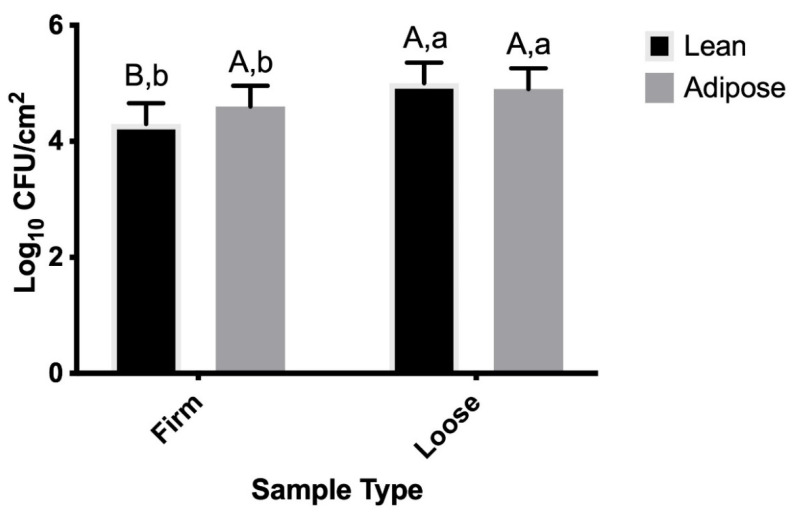
Populations of Shiga toxin-producing *Escherichia coli* (STEC) cells grown in tryptic soy broth (TSB) represent loose and firm attachment to lean and adipose beef brisket tissues within 20 min post-inoculation. Sample type × tissue type was significant (*p* < 0.0001). As the time × sample type × tissue type interaction was not significant (*p* > 0.05), data for each time point are combined. ^A,B^ Observations with different superscripts within sample type vary statistically (*p* ≤ 0.05). ^a,b^ Observations with different superscripts within tissue type vary statistically (*p* ≤ 0.05).

The main effect of sample type (*p* < 0.0001) and the sample type × tissue type (*p* = 0.0052) interaction were significant within the first 20 min for TSB-grown STEC; however, because sample type is included in the interaction, sample type data will only be shown as it pertains to tissue type. Within the first 20 min, more TSB-grown STEC firmly attached to adipose tissue than to the lean tissue (*p* = 0.0020); however, the difference in populations was a modest 0.2 log CFU/cm^2^ (Figure 4). The largest difference in population (0.6 log CFU/cm^2^) was observed between firmly attached and loosely attached STEC on lean tissues (*p* < 0.0001).

STEC cells grown in M9 did not demonstrate any significant main effects or interactions (*p* > 0.05) within the first 20 min post-inoculation. Thus, temperature of the beef tissue was not a significant variable for M9-grown STEC attachment within the first 20 min post-inoculation.

## 4. Discussion

### 4.1. TSB-Grown STEC

Longer durations of storage often result in increased attachment rates, particularly when duration shifts from hours to days [10]. TSB-grown STEC become more firmly attached to beef as time progresses (Table 1), which is consistent with previously published findings. Figure 1 illustrates that STEC more firmly attach to adipose tissue than lean tissue and, subsequently, a larger population of loosely attached STEC was recovered from lean beef tissue. While it is expected that adipose tissue would be less hydrophobic, a study examining the ability of *S.* Typhimurium to attach to chicken surfaces concluded that damaged fatty cells may result in a “fatty coating” that creates an enhanced hydrophobic surface, resulting in an increase of bacterial adhesion to beef tissue [25]. In the present study, the greatest difference in population was 0.3 log CFU/cm^2^, which was detected between STEC firmly attached versus loosely attached to lean tissue (*p* < 0.0001). While this difference is statistically significant, it is not of great magnitude from a biological sense. In general, the TSB-grown STEC data are in agreement with the literature that STEC attachment is similar on lean and adipose beef tissue [15,18,24,25].

Previously published research indicates that other pathogens behave similarly to STEC when attaching to lean and adipose beef tissues. *Salmonella choleraesuis* better adhered to lean versus adipose tissue, although the difference in attachment was less than 0.4 logs [31]. A survey of attachment involving *Serratia marcescens*, *Staphylococcus aureus*, *Streptococcus faecalis*, *Salmonella arizonae*, *Pseudomonas aeruginosa*, and *Listeria monocytogenes* to lean and adipose tissue showed no significant difference in populations for any of the organisms [16]. Contaminating beef tissue by cattle manure inoculated with *Salmonella* Typhimurium and *L. monocytogenes* resulted in similar attachment patterns between lean and adipose tissue and population differences were insignificant or less than 0.5 logs [17].

### 4.2. M9-Grown STEC

Figure 2 illustrates that the population of M9-grown STEC was variable throughout the 2880 min attachment period. The largest population of M9-grown STEC was recovered from the 2880 min (48 h) time point (4.1 log CFU/cm^2^), suggesting that STEC populations eventually grew towards the end of storage. The M9-grown STEC underwent consecutive stressors: nutrient limitations in M9 media followed by cold (4 °C) storage temperature, and this “double stress”, followed by the selectivity of MacConkey agar used for enumeration, may have impacted populations. Therefore, it is possible that the increased population at 2880 min (48 h) occurred as a result of STEC acclimating to, and overcoming, the combination of stressful environments. It is expected that the refrigeration temperature would hinder the growth of STEC, as previous research has demonstrated that refrigeration extends the length or presence of lag phase growth [12]. Thus, rather than definitively concluding that STEC grew during storage at 4 °C, it should also be considered that this discrepancy in population might have been the result of difficulty enumerating injured/stressed cells prior to the 2880 min sampling point. It is also important to recognize that the variability was within ± 0.5 log CFU/cm^2^ of the 0 min time point throughout the study. Therefore, although significant differences were detected, these differences were small in magnitude, which must also be considered in terms of biological relevance.

Figure 3 illustrates that larger populations of M9-grown STEC attached to adipose tissue than lean tissue. However, the difference in attachment was 0.2 log CFU/cm^2^, which is negligible from a biological sense. Therefore, although they are statistically different, these data are not particularly informative in regard to understanding M9-grown STEC attachment on beef tissues.

When bacteria are grown in “starvation stress” media, the population of bacteria (*Listeria monocytogenes*, *Salmonella typhimurium*, and *E. coli* O157:H7) attached to lean and adipose beef tissue decreased in some studies [18]. Cells that are able to survive starvation have exhibited a decreased ability to attach to beef, although not significantly [18], and cellular stress induced by such a growth medium (such as a minimal salt medium) may influence cellular attachment [32]. Similarly, it has been documented that manipulation of growth conditions can affect STEC attachment to surfaces such as stainless steel [33], and the present study suggests this is also true for STEC attachment to beef tissue surfaces. Although attachment of TSB- and M9-grown STEC were not statistically compared, it was generally observed that M9-grown STEC attachment was more inconsistent than TSB-grown STEC, which supports previously published studies that cellular stress influences bacterial cell attachment. Growing STEC cells in M9 medium was intended to replicate the stress that STEC undergo on the hide, and/or during beef processing and fabrication, which may provide a better representation of how STEC might attach in real-world scenarios.

Recent studies reporting on STEC attachment to abiotic surfaces suggest that characteristics of the attachment surface may influence affinity of STEC cells for attachment more so than media type. When grown in M9 and Luria Bertani (LB), *E. coli* O111 and O45 demonstrated strong biofilm development on a polystyrene surface [34] but lacked biofilm development on stainless steel [35]. Unlike Wang et al. [34] and Ma et al. [35], the present study did not investigate biofilms or abiotic surfaces, and these variations in methodology likely contributed to these differences.

### 4.3. Influence of Beef Tissue Temperature on STEC Attachment

Data collected from time points 0 min through 2880 min (48 h) suggest that temperature of the beef tissues did not impact TSB- or M9-grown STEC attachment to adipose or lean tissue beef surfaces. The impact of temperature may have been confounded by the experimental design, as all warm samples were placed into refrigerated storage at 30 min post-inoculation, while all cold samples were maintained at 4 °C throughout the study. Separate statistical analyses were conducted for data collected from time points 0 min through 20 min (all samples were refrigerated 30 min post-inoculation) in order to address this potentially confounding factor, and temperature (warm versus cold samples) was not a significant variable (*p* > 0.05), nor was it included in any significant interactions. Thus, it can be concluded that initial temperature of either tissue type did not impact STEC attachment within the first 20 min in this study. Similarly, the same sample type × tissue type significant interaction was observed for the first 20 min of TSB-grown STEC attachment (Figure 4) as was observed for TSB-grown STEC attachment throughout the full 2880 min (Figure 1).

Some studies have reported mixed results regarding the impact of temperature on bacterial attachment. For example, researchers have shown that growth temperatures may impact attachment on adipose tissue but not lean tissue [24]. When *E. coli* O157:H7 cells were held under nutrient-limiting conditions at various temperatures, attachment decreased as storage temperature increased, although not to a statistically significant level [18]. In another study, TSB-grown STEC attachment was significantly higher on chilled brisket in comparison to non-chilled brisket; however, the difference was marginal at 0.4 log_10_ CFU/cm^2^ [22]. In the present study, STEC attachment was not impacted by temperature of the beef tissue, suggesting that TSB- and M9-grown STEC attach similarly to pre-chill and post-chill beef carcasses within the first 20 min of contamination.

## 5. Conclusions

Previous research primarily 1) investigated the temperature at which bacteria were grown or stored to assess the impact of temperature on bacterial attachment, and 2) focused on *E. coli* O157:H7, or other pathogens, using both nutrient-dense and nutrient-limited culture methods. The present study contributes new knowledge to this body of evidence by simulating pre-chill and post-chill carcass attachment to probe the impact of carcass temperature on attachment of *E. coli* O157:H7, O26, O45, O103, O111, O121, and O145, grown in both nutrient-dense and nutrient-limited media.

This study demonstrated that firmly attached STEC cells increase throughout time, especially when STEC cells originate from TSB. STEC cells originating from M9 displayed variable attachment, which suggests that the metabolic state of STEC influences the ability of cells to adhere to beef surfaces. In general, these data are in agreement with previously published research describing bacterial attachment to beef surfaces.

While a body of evidence currently exists on how factors like temperature, stress, and tissue type impact bacterial attachment, the present study is unique in that it incorporates all of these factors to understand how a cocktail of *E. coli* O157:H7 and the “Big Six” adulterant serogroups attach during simulated beef harvest and postharvest processes. On that note, it should also be mentioned that this study has several limitations. First, because a cocktail was used, these data cannot be used to understand attachment for individual strains or serogroups, and a study investigating individual strains or serogroups could be the focus of future research. Second, this was a laboratory-controlled study designed to simulate STEC attachment to beef carcasses using briskets and temperature-controlled incubators. Therefore, these data are very preliminary in nature and additional research is necessary to fully understand STEC attachment to beef carcasses at the abattoir.

Further understanding of the effect of the media of origin, and thus the environment immediately preceding contamination, is needed before data can be effectively used for future food safety practices at the abattoir. This is an important variable to consider, as bacterial cells, including STEC, that are entering the abattoir on the hide of an animal are not experiencing the same environmental conditions that optimal growth parameters typically used in the laboratory setting would provide. The attachment data described herein can inform future investigations designed to evaluate the possibility of reduced intervention efficacy of STEC on post-chill and subprimal cuts of beef.

## Figures and Tables

**Table 1:** Populations of Shiga toxin-producing *Escherichia coli* (STEC) cells grown in tryptic soy broth (TSB) represent loose and firm attachment to beef brisket tissues during 48 h (2880 min) of storage at 4 °C. Time × sample type was significant (*p* < 0.0001). As the time × sample type × tissue type interaction was not significant (*p* > 0.05), data for adipose and lean tissue attachment are combined for each data point.

	Sample Type	
Time (min)	Loose (log CFU/cm^2^)	Firm (log CFU/cm^2^)
0	4.9 ^a,A^	4.4 ^a,B^
3	4.9 ^a,A^	4.6 ^b,c,B^
5	4.9 ^a,A^	4.4 ^a,B^
20	4.9 ^a,A^	4.4 ^a,B^
60	4.6 ^b,A^	4.5 ^a,b,A^
180	4.4 ^b,c,A^	4.6 ^a,b,A^
480	4.4 ^b,c,A^	4.5 ^a,b,A^
720	4.3 ^c,A^	4.5 ^a,b,B^
1440	4.3 ^c,A^	4.6 ^b,c,B^
2880	4.3 ^c,A^	4.8 ^c,B^

^a,b,c^ Observations with different superscripts within a column vary statistically (*p* ≤ 0.05). ^A,B^ Observations with different superscripts within a row vary statistically (*p* ≤ 0.05).

## Data Availability

The data presented in this study are available upon request from the corresponding author.
